# Modelling temperature and humidity effects on web performance: implications for predicting orb-web spider (*Argiope* spp.) foraging under Australian climate change scenarios

**DOI:** 10.1093/conphys/coz083

**Published:** 2019-12-08

**Authors:** S J Blamires, W I Sellers

**Affiliations:** 1 Evolution & Ecology Research Centre, School of Biological, Earth and Environmental Sciences, The University of New South Wales, Sydney, New South Wales 2052, Australia; 2 School of Earth and Environmental Sciences, The University of Manchester, Williamson Building, Manchester M13 9PL, UK

**Keywords:** Climate change projections, extended phenotype, humidity effects, multibody dynamic model, spider web function, temperature effects

## Abstract

Phenotypic features extending beyond the body, or EPs, may vary plastically across environments. EP constructs, such as spider webs, vary in property across environments as a result of changes to the physiology of the animal or interactions between the environment and the integrity of the material from which the EP is manufactured. Due to the complexity of the interactions between EP constructs and the environment, the impact of climate change on EP functional integrity is poorly understood. Here we used a dynamic model to assess how temperature and humidity influence spider web major ampullate (MA) silk properties. MA silk is the silk that absorbs the impact of prey striking the web, hence our model provides a useful interpretation of web performance over the temperature (i.e. 20–55°C) and humidity (i.e. 15–100%) ranges assessed. Our results showed that extremely high or low humidity had direct negative effects on web capture performance, with changes in temperature likely having indirect effects. Undeniably, the effect of temperature on web architecture and its interactive effect with humidity on web tension and capture thread stickiness need to be factored into any further predictions of plausible climate change impacts. Since our study is the first to model plasticity in an EP construct’s functionality and to extrapolate the results to predict climate change impacts, it stands as a template for future studies that endeavour to make predictions about the influence of climate change on animal EPs.

## Teaser

Lay Summary.

How climate change impacts animal extended phenotypes (EPs) is poorly understood. We modelled how temperature and humidity affects the ability of spider webs to intercept prey. We found humidity had negative effects at the extremes. Temperature, however, likely interacts with humidity to affect web tension and prey retention.

## Introduction

Animal phenotypic features, whether they are morphological, biochemical or behavioural features, can vary plastically across environments as a consequence of the interplay between genetic and environmental influences (Schneider, 1993; Sih *et al*., 2004; Borges, 2008). It is generally thought that plasticity in specific traits enhances Darwinian fitness by enabling an organism to retain critical functionality across environments (Sarkar and Fuller, 2003; Borges, 2008). Some organisms construct phenotypic features ‘beyond the body’ (Turner, 2000; Waite and Broomell, 2012). These features have been called ‘external organs’ (Turner, 2000) or, more commonly, ‘extended phenotypes’ (EPs) (Dawkins, 1982; Blamires, 2010; Bailey, 2012). EPs are not only physical constructs, such as spider webs, beaver dams, midge-induced plant galls, worm burrows and termite mounds, but anything that exerts indirect genetic effects (Bailey, 2012). These include animal signals, the manipulation of a host by ectoparasites and other intraspecific and interspecific interactions (Dawkins, 1982; Wang *et al*., 2008; Schaedelin and Taborsky, 2009; Hoover *et al*., 2011; Fisher *et al*., 2019). We use the term EP constructs herein to differentiate physical constructs from all other forms of animal EPs. While it is acknowledged that EP constructs plastically vary across environments because of an interplay between genetic and environmental influences (Blamires, 2010; DiRienzo and Montiglio, 2016; Blamires *et al*., 2018; DiRienzo and Aonuma, 2019), how plasticity in EP constructs affect the fitness of organisms across environments is not well known (Bailey, 2012; Fisher *et al*., 2019).

EP constructs produced from secreted biomaterials are adapted to perform a specific function, or functions, under certain conditions (Waite and Broomell, 2012; Blamires *et al*., 2018). The functional optimality and limitations of the materials nevertheless are often environment specific. For instance, it has been deduced that the adhesive performance of spider orb web gluey silks are, for any given species, optimally adhesive at the humidity most commonly experienced by the secreting spider (Opell *et al*., 2018).

Temperature and humidity are environmental variables known to affect the mechanical performance of somatic phenotypic traits, such as stimulation of motor nerves, and muscle activation and relaxation time in fish and frogs (Johnston and Temple, 2002; Bennett and Lenski, 2007; Frazier *et al*., 2008). As global temperatures and humidities change as a result of climate change (Webb and Hennessy, 2015), adapting to future climate scenarios presents as a serious challenge for many animals (Krehenwinkel and Tautz, 2013; Merilä and Hendry, 2014). The specific challenges vary across geographic regions. Around Sydney, Australia, for instance, annual temperatures are predicted to increase by almost 5°C by 2090 under the most extreme climate change scenarios. This will be manifested as more days (11 up from 3 at present) per year exceed 35°C. Meanwhile, mean spring rainfall is expected to reduce by as much as 34%. All the while, relative humidity will decrease by up to 4% across all seasons, and evapotranspiration will increase by as much as 18% (Webb and Hennessy, 2015). Animals of the region will thus need to cope with a significantly hotter and drier climate.

The influence of temperature and/or humidity on the performance of EP constructs is nonetheless not well understood, rendering it extremely difficult to make meaningful predictions about the impacts of climate change on the fitness of the animals that build them. Temperature might affect the biomechanical properties of EP constructs in one or both of the following ways: (i) exceeding the builder’s upper thermal tolerance limit, i.e. its critical thermal maximum, thus impeding its ability to produce the materials from which the EP is constructed and/or to fashion the materials into a functional construct; (ii) directly affecting the structural integrity of the EP and/or the molecular integrity of the material from which it is manufactured (Alam *et al*., 2007; Bailey, 2012; Waite and Broomell, 2012).

Spider orb webs are characterized as having a two-dimensional circular-shaped capture area containing a single sticky capture thread spiralling outward from the hub and evenly distributed radial threads that span from the hub to the web periphery (Foelix, 2011). They are the most intriguing and commonly examined EP constructs among researchers aiming to understand the plastic responses of EPs (Blamires, 2010; Nakata, 2012; Blamires *et al*., 2017a, 2018; DiRienzo and Aonuma, 2019). This is because they are constructed from a unique biomaterial, silk, which is specifically adapted to perform the function of capturing and retaining insects in full flight (Blackledge and Hayashi, 2006; Sensenig *et al*., 2013; Blamires *et al*., 2017b). Direct consequences on fitness are thus expected when web properties vary across environments (Blamires *et al*., 2018).

Each of the seven or so silks secreted by orb-web-building spiders is incorporated into their webs, and each has distinct properties (Blamires *et al*., 2017b). The most impressive of which are those of major ampullate (MA) silk. The role of this silk is to intercept insects, or on occasions, birds or bats (Nentwig and Heimer, 1987; Nyffeler and Knornschild, 2012), in full flight by absorbing and dissipating the exorbitant amounts of kinetic energy imparted into the web (Craig, 1987; Sensenig *et al*., 2012, 2013; Harmer *et al*., 2015).

Exposure to ecologically high temperature and low humidity induces mechanical property changes in MA silk, with its strength and stiffness increasing with temperature (Blamires *et al*., 2012). However, the functional consequence of different temperatures on spider webs has never been empirically determined. This is, by and large, because of the complexity of interplaying variation in the following: (i) the web building, silk spinning and locomotor behaviours of the spider (Guess and Viney, 1998; Vollrath *et al*., 2001; Moore *et al*., 2016); (ii) the spider’s responsiveness to web stimulation; (iii) flight paths and performance of the insects (Frazier *et al*., 2008); (iv) the web’s architectural features (Vollrath *et al*., 1997); and (iv) the interactive properties of each of the silks incorporated into the web (Blamires *et al*., 2012). Thus, performing controlled experiments are rendered highly problematic.

One study (Boutry and Blackledge, 2013) has examined the influence of humidity on spider web prey capture performance. It showed that webs at high humidity (>70% RH) intercepted prey better without breaking than did those at low humidity (30–35% RH). The authors attributed this finding to MA silks within the web’s radials shrinking and becoming more compliant (a phenomenon called supercontraction) thus dissipating more kinetic energy at impact. In addition to providing a mechanism for tensioning webs, supercontraction at high humidity might counteract web stiffening at ecologically high temperatures in tropical orb webs. Spiders constructing webs in Sydney in the summer of 2090 will, nevertheless, likely be exposed to extremely high temperatures and low humidity. It might accordingly be expected that their prey capture performance will become critically impaired compared with that of today’s spider webs.

Simulations using finite element, dynamic and analogous models are becoming increasingly utilized for resolving complex problems and making detailed predictions in biological engineering based on the properties of the structural elements (Stachurski, 1987; Alam *et al*., 2007). There are two modelling approaches generally used to model the performance of spider webs. The most common is finite element structural analysis (Alam *et al*., 2007; Harmer *et al*., 2015), and this works well for modelling the material deformation and contact interactions. However, an alternative approach is multibody dynamic analysis (Tarakanova and Buehler, 2012), which allows for incorporation of larger scale movements, such as the trajectory of the projectile, and momentum/energy transfer over longer time periods.

Here we used a multibody dynamic model that uses reported properties of spider MA silk to test whether the ability of spider orb webs to capture prey are likely to become affected by climate change. We created simulations of a projectile (representing a flying insect) hitting a web and varied the material properties of the web over a temperature range of 20–55°C and a humidity range of 15–100%. The outputs of the model across the temperature and humidity ranges tested were then used to make predictions about the likely future web performances of summer active spiders from around Sydney under extreme climate change scenarios.

## Materials and Methods

For our dynamic model, we used the mean web geometric parameters reported for ‘large frame’ webs built by the orb-web spider *Argiope radon* (Harmer *et al*., 2015). We selected this species because it has the most complete and, accordingly, most reliable data set available for modelling purposes (Table 1; Harmer *et al*., 2015), and it is closely related to the most common summer-active orb-web spider from Sydney, *Argiope keyserlingi*. However, it is important to acknowledge that there are many studies on the material properties of various spider silks, and the values reported may vary considerably. For example, Blamires *et al*. (2012) collected the values for MA silk for 10 orb-web species from Taiwan and showed a variation of 232–909 MPa for ultimate strength, 47–202 MJ m^−3^ for toughness and 3.4–10.7 GPa for Young’s modulus (Blamires *et al.*, 2012). These value ranges might mask the variation found within a single web, e.g. 2.6–7.0 GPa for the Young’s moduli of radial, frame and mooring threads (Alam *et al.*, 2007), as well as likely variation due to methodological differences and collection protocols, for instance between forcible silking and web-collected silks (Blamires *et al.*, 2012, 2017b).

**Table 1:** Geometric properties of the web based on those measured for *A. radon* (Harmer *et al*., 2015).

Geometrical parameter	Size (m)
External radius	0.183
Internal radius	0.011
Support width	0.5
Support height	0.5
Spiral pitch	0.004
Spiral turns	43
Radial strands	31

The model required the input of the following silk material properties: thread diameter, elastic modulus, breaking strain and initial tension (Lin *et al*., 1995; Alam *et al*., 2007). The parameter values chosen were those originally reported by Lin *et al*. (1995), who used a combination of high-speed videometry of pallets fired at a web and Finite Element modelling (Table 2). To calculate the web mass distribution, a mean silk density of 1250 kg m^3^ (Laity *et al*., 2015; Ko and Wan, 2018) was used.

**Table 2:** Material properties of the orb web based on common MA silk property values (Alam *et al*., 2007).

Type of threads	Diameter of threads (μm)	Modulus of elasticity (MPa)	Initial tension (μN)	Breaking strain
Spiral	2.40	500	10	1.600
Radial	3.93	2600	132	0.462
Frame	7.23	5555	924	0.225

**Figure 1:**
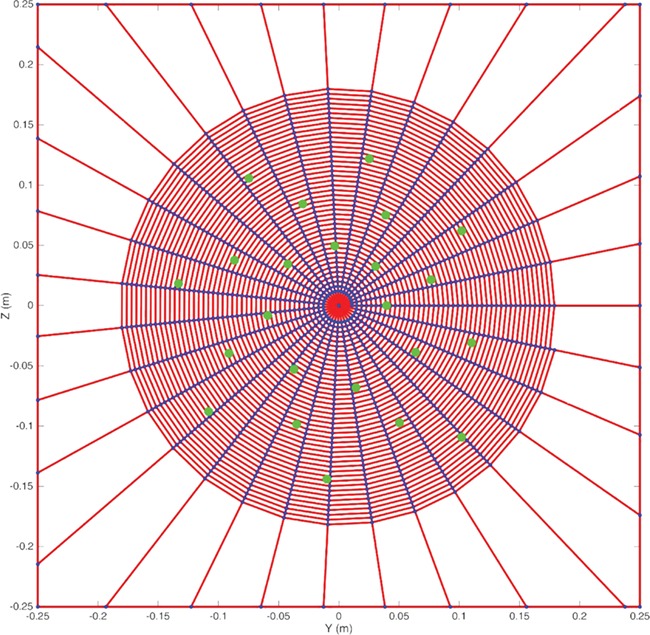
The web geometry created based on those measured for *A. radon* (Harmer *et al*., 2015). Twenty-three target locations for projectile impact were also defined on a regular pattern to cover the web surface as illustrated. The model consists of 31 radial threads and a single spiral thread that makes 43 turns, as specified in Table 1. The web is suspended in a square frame. The model consists of 2731 links, made up of 43 + 2×31 radial links, 43×31−1 spiral links and 4 frame links.

Experimental work has demonstrated the effectiveness of using artificial projectiles to assess the prey capture abilities of different webs (Sensenig *et al*., 2012, 2013), and similar approaches have been used in previous simulation studies (Harmer *et al*., 2015). We therefore adopted this approach using a projectile with a mass of 500 mg and a horizontal impact velocity of 2.0 m s^-1^, therefore an impact kinetic energy of 1.0 mJ. The impact was tested at 23 different points on the web, as illustrated in Fig. 1. To account for web property changes across temperatures and humidities, we altered the stiffness and breaking strains in line with the values obtained experimentally for another species of *Argiope* (Plaza *et al*., 2006). These values are only indicative of possible property changes that might be perceived, since it is very likely that the different silk formulations within the web react differently and there is likely to be considerable differences across spider species (as indeed there are for all material and geometric properties of spider webs). The typical ranges that might be predicted are based on varying the humidity from 15% to 100% at a constant temperature (i.e. 55°C), which are shown in Table 3. Similar data can be used to estimate the range of effects for temperature at a constant humidity (i.e. 50%) (Table 4). It is noted that the temperature and humidity ranges used herein are outside the range that spider webs are likely to encounter, even under worst-case scenario climate change projections (Schneider, 2009). Nevertheless, the ranges used are illustratively very useful. The percentage changes in Tables 3 and 4 were then applied to the material properties in Table 1 to provide the following test scenarios: low (−78% max strain, +94% mean stiffness), medium and high (+78% max strain, −94% mean stiffness) humidity; and low (+1% max strain, +18% mean stiffness), medium and high (−1% max strain, −18% mean stiffness) temperature.

**Table 3:** Effects of humidity on the material properties of *Argiope trifasciata* MA gland silk fibres (Fig. 2A; Plaza *et al*., 2006).

Humidity	Max strain	Mean stiffness (MPa)
15%	0.246	4170
50%	0.270	3440
70%	1.059	680
100%	1.953	140
Range	±78%	±94%

**Table 4:** Effects of temperature on the material properties of *A. trifasciata* MA gland silk fibres (Fig. 2B; Plaza *et al*., 2006).

Temperature (°C)	Max strain	Mean stiffness (MPa)
20	0.264	5280
55	0.257	3640
Range	±1%	±18%

## Results

**Figure 2:**
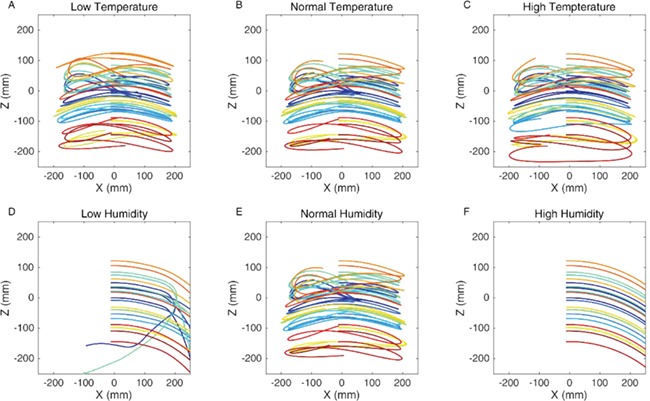
Trajectories of the projectile after impact for the various climatic conditions are as follows: low temperature (+1% max strain, +18% mean stiffness) (**A**), medium temperature (**B**) and high temperature (−1% max strain, −18% mean stiffness) (**C**) and low humidity (−78% max strain, +94% mean stiffness) (**D**), medium humidity (**E**) and high humidity (+78% max strain, −94% mean stiffness) (**F**).

The simulations were run for a 500-ms duration from a position where the projectile was a few millimetres from contact with the web. Fig. 2 shows the forward and vertical components of the trajectory following impact with the web. The ability of the web to stop the projectile is not impaired across the different temperature scenarios, but under high humidity, the projectile passes through the web in all cases and in almost all cases under low humidity. These results show that in this case, temperature alone has little effect on prey capture, but that the effect of humidity can be extreme with both low and high humidity having potentially deleterious effects on prey capture.

**Figure 3:**
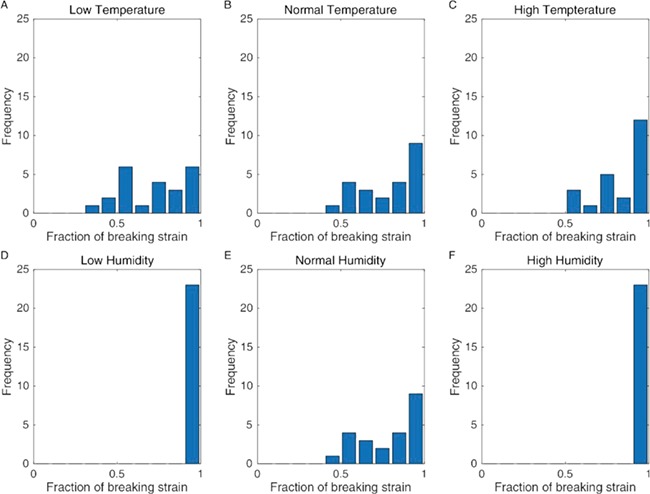
Maximum strains recorded as a fraction of the breaking strain for the individual web components for the various climatic conditions are as follows: low temperature (+1% max strain, +18% mean stiffness) (**A**), medium temperature (**B**) and high temperature (−1% max strain, −18% mean stiffness) (**C**) and low humidity (−78% max strain, +94% mean stiffness) (**D**), medium humidity (**E**) and high humidity (+78% max strain, −94% mean stiffness) (**F**).

**Figure 4:**
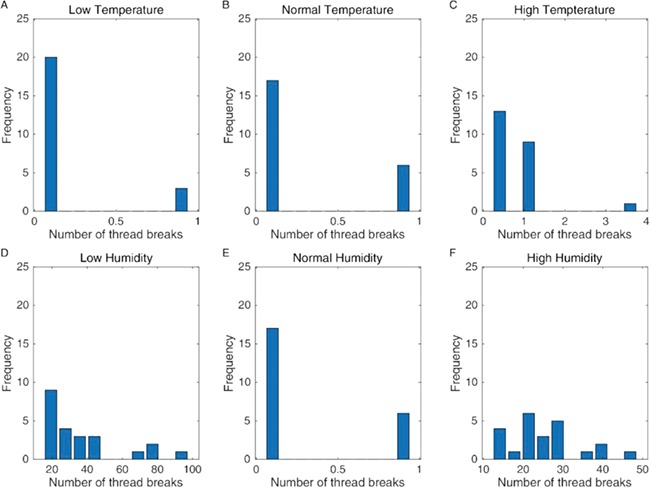
The numbers of individual threads broken for the various climatic conditions are as follows: low temperature (+1% max strain, +18% mean stiffness) (**A**), medium temperature (**B**) and high temperature (−1% max strain, −18% mean stiffness) (**C**) and low humidity (−78% max strain, +94% mean stiffness) (**D**), medium humidity (**E**) and high humidity (+78% max strain, −94% mean stiffness) (**F**).

However, prey capture is not the only aspect of web functionality that we are concerned with here. Prey impact also causes damage to the web, and the spider must spend both time and energy repairing the damage. Our model was also able to calculate the stresses acting on the individual threads. Fig. 3 shows how the distribution of thread stresses is affected by the changing material properties of the thread in each of the temperature and humidity scenarios. In all situations, the projectile can lead to web damage depending where the web is hit, but the distribution is much more left skewed (i.e. towards 1) in both the high- and low-humidity scenarios. In Fig. 4, we show the actual number of broken threads generated by the 23 impact cases for the different climatic conditions and support the suggestion (Boutry and Blackledge, 2013) that lowering the humidity leads to both significant web damage and reduced prey capture.

**Figure 5:**
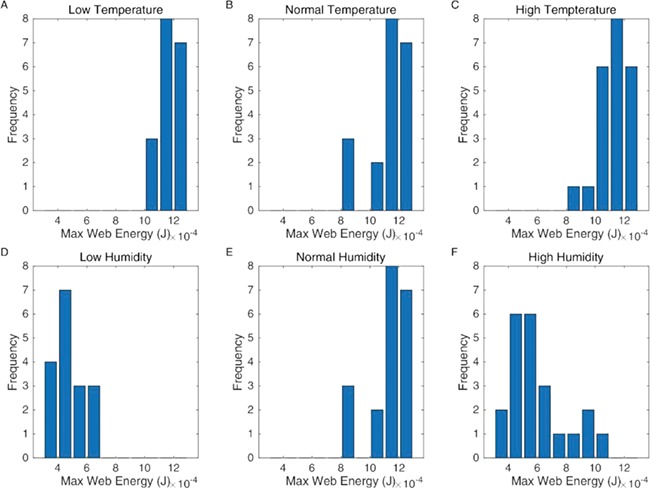
Maximum strain energy stored in the web for the various climatic conditions are as follows: low temperature (+1% max strain, +18% mean stiffness) (**A**), medium temperature (**B**) and high temperature (−1% max strain, −18% mean stiffness) (**C**) and low humidity (−78% max strain, +94% mean stiffness) (**D**), medium humidity (**E**) and high humidity (+78% max strain, −94% mean stiffness) (**F**).

The reasoning behind the predicted changes in web performance is demonstrated in Fig. 5. Here we plotted the maximum strain energy of the web and showed that in the cases where prey capture is effective, the web is able to contain the 1 mJ kinetic energy of the projectile. Whereas in cases where prey capture is ineffective, the peak energy is much lower since the forward velocity of the projectile is not entirely removed. Again, the high-humidity scenario is the worst performing, although the low-humidity scenario also performed poorly. In many cases, the energy transferred to the web is marginally <1 mJ because the projectile never reaches a standstill in any of these simulations. Moreover, there is also gravity and damping interactions that may have additional effects on energy partitioning.

## Discussion

A broad body of work has shown spider webs and silks to have considerable architectural and functional plasticity as temperature, humidity, Ultraviolet (UV) exposure and the number and types of predator and prey species present vary (Vollrath *et al*., 1997; Heiling and Herberstein, 2000; Blamires *et al*., 2007; Blamires, 2010; Blackledge *et al*., 2011; Boutry and Blackledge, 2013; Blamires *et al*., 2016, 2017a). Moreover, experiments have shown that such plasticity can influence the fitness of spiders via tuning of the web’s ability to capture and retain prey across different environments (Blackledge *et al*., 2011; Harmer *et al*., 2011; Opell *et al*., 2017; Blamires *et al*., 2018). However, until now no study has used the knowledge ascertained to make any predictions about how the performance of spider webs copes under climate change scenarios.

We used here a multibody dynamic model testing the ability of orb webs of *Argiope* spp. to absorb the impact kinetic energy of a theoretical flying prey across a 20–55°C temperature range and a 15–100% humidity range. We used some common MA silk property values to estimate web performance across the temperature range. We consider this approach appropriate as MA silk is the toughest silk within the orb web to provide the majority of the web’s structural integrity (Harmer *et al*., 2011). Moreover, it is the silk that absorbs all of the impact of an insect striking the web (Craig, 1987; Sensenig *et al*., 2012, 2013; Harmer *et al*., 2015). Thus, if it fails insects cannot be captured, let alone retained, by a web.

We found measurable effects for all the parameter manipulations we tried. The effects of temperature alone were relatively small as would be expected from the small percentage changes in material properties they would be expected to generate. However, because both breaking strain and mean stiffness decrease at high temperatures (Guess and Viney, 1998), these factors would act together to reduce the overall toughness of the material. While prey capture success rates are not affected, web damage is greater with higher temperatures leading to higher strains as a proportion of breaking strain and a higher number of thread breakages. The effects due to humidity were nevertheless more prominent, as predicted from the much larger numerical effects on the material properties inputted. Because the effects of temperature and humidity on stiffness and breaking strain are in opposite directions, we might expect a complex picture to emerge. This is indeed the case, and we see that dramatic shifts in both factors lead to much poorer web performance. Webs at low humidity were projected to have a poor capture performance, although the high-humidity scenario also performed poorly with most projectiles passing through the web. The poor capture performance at low and high humidity leads to extensive web damage with marginally worse performance under the low-humidity scenario. The reduced stopping success of the web is clearly due to the reduced strain energy capacity of the silks due to the alterations in their stiffness and breaking strain. These predictions, however, depend on the precise values used in the model and, as noted earlier, there is considerable variation in the values reported in the literature. The values used herein, taken from Plaza et al. (2006), show an 8-fold increase in the maximum strain and a 30-fold reduction in the mean stiffness over the range of humidities they tested (Table 3). With a linear model, this means that the toughness reduces by a factor of 3.8. However, data on the effects of silk supercontraction at high humidities suggest that in some situations, the overall toughness (hence resistance to breaking under prey impact) increases (Boutry and Blackledge, 2013). Interestingly, Vehoff *et al*. (2007) showed a 1.2-fold reduction in toughness for *Nephila senegalensis* at high humidity but a 1.3-fold increase for *Nephila clavipes*, illustrating the complex nature of the interaction between stiffness and breaking strain even among closely related species. These discrepancies all illustrate the difficulties of trying to generalize from a single model and the problems of using inconsistent data sources as well as modelling spider silk as a linear spring. Ideally, all the required modelling parameters should be measured in a uniform fashion for a range of species with uncertainties and variation explored using sensitivity analysis (Campolongo *et al*., 2000), but this is a major undertaking and beyond the scope of this paper.

The temperature range used in our models exceeded even the worst-case scenario climate change projections (Schneider, 2009). Nonetheless, we considered it necessary to fully assess the dynamics of web property variability as temperatures rapidly change over a wide range to be able to predict the values that pertain to actual climate change scenarios. For instance, in and around Sydney, extreme climate change predictions have annual temperatures increasing by ~ 5°C by 2090, while humidity is predicted to decrease by ~ 4%RH (Webb and Hennessy, 2015). More significantly, the number of days per year that exceeds 35°C will profoundly increase (Webb and Hennessy, 2015). Our modelling herein, along with previous work (Blamires *et al*., 2012), has shown that radial threads become brittle and liable to easily break when exposed to temperatures over 35°C for several consecutive days. If extreme climate change scenarios are realized, then the functionality of *Argiope* webs will undoubtedly be negatively affected. Given that the particular species for which our model was developed (*A. radon* and *A. keyserlingi*) are broadly distributed throughout Australia, any populations outside of Sydney may experience different climate change scenarios which could influence the properties of these spider’s webs differently.

Extreme climate change predictions posit a decrease in humidity of ~4% RH in and around Sydney (Webb and Hennessy, 2015). Our model showed that web function is negatively affected under extreme high- or low-humidity scenarios. We nonetheless do not expect a fall of 4% RH to adversely affect the functionality of spider webs in Sydney. Notwithstanding, the functionality of *Argiope* webs is improved if they are built at high (>70%) humidity (as occurs during the morning or evening), as supercontraction of the radial silks enhances the web’s tension and compliance, hence its capacity to absorb the kinetic energy imparted by flying prey (Boutry and Blackledge, 2013). It thus may be reasonable to predict that the performance of *Argiope* webs might be detrimentally impacted by climate change if the morning and/or evening humidity decreases substantially during the summer.

It is important to note that while we used data from several sources (e.g. Lin *et al*., 1995; Plaza *et al*., 2006; Alam *et al*., 2007; Boutry and Blackledge, 2013; Harmer *et al.*, 2015), the models presented here are just examples of the broader findings that might be expected. We used some common material and web geometry values rather than any specified values for a given spider species in a specific environment. There is a great deal of variation in all the parameters that make up the model, and to properly evaluate the effects on a particular species would require us to produce a set of species and environment-specific cases. There is nevertheless scope for increasing the realism of the model. The material properties of real spider silks are complex, and the effects of moderate environmental changes are largely unknown. Added to this complexity is the fact that orb webs are rarely constructed as a uniform plane, and the properties such as web size, mesh height and the number of, and angles between, radial threads are not the same across different web sectors (Blackledge *et al*., 2011; Soler and Zaera, 2016). Moreover, the impacts that prey impart on webs are more complex than a simple projectile with directed variations in speed, direction, mass and surface area. There are important effects imparted by aerodynamic forces as well as complex speed dependent stress-strain relationships for the individual silks that make up the web. In addition, because of the non-linear nature of MA silk, a thread either breaks if its breaking strain is exceeded or it does not if the breaking strain is not exceeded. We thus potentially have multiple cliff-edge scenarios when even small changes may lead to catastrophic effects in some situations. All factors accordingly need to be properly explored further to fully quantify the probable effects of particular climate change scenarios.

The mechanical performance of the MA silks within spider orb webs are non-linear, i.e. they undergo initial softening up to a yield point whereupon the silk substantially stiffens until reaching a modestly large strain of failure (Cranford *et al*., 2012). Indeed, the dynamic non-linear material property of MA silk is identified as a critical aspect of the web’s ability to intercept prey (Blackledge *et al*., 2011; Cranford *et al*., 2012). Similarly, the geometrical properties of the web, such as fibre diameter, are not uniform and likely to be functionally important (Jyoti *et al*., 2018). We used a multibody dynamic model here rather than Finite Element Analysis (FEA) (Harmer *et al*., 2015), as it copes with the non-linear, large strain properties of silk better than FEA (Tarakanova and Buehler, 2012). Moreover, web pretension can be adjusted to account for the compounding effects of web tension under different temperature and humidity scenarios in addition to the silk’s impact absorption properties. Again, supercontraction may be important since there is evidence that it greatly increases web tension (Savage *et al*., 2004; Boutry and Blackledge, 2013).

Our simulations repeatedly showed humidity to significantly affect the impact absorption capabilities of spider orb webs while temperature made little difference. Absorbing the impact of flying prey is nevertheless the first of a series of actions within the web that affects whether spiders can capture and retain certain prey. The retention of prey intercepted, and their ultimate recognition by the spider are equally, or perhaps more, important actions (Blackledge *et al*., 2011; Tarakanova and Buehler, 2012; Blamires *et al*., 2018). Prey retention is driven by the adhesiveness of the gluey silks of the spiral threads (Craig, 1987; Tarakanova and Buehler, 2012; Blamires *et al*., 2018), which are dynamically affected by the interplay between temperature and humidity (Stellwagen *et al*., 2014; Opell *et al*., 2017), as well as UV radiation (Stellwagen *et al*., 2015, 2016). It is well known now that at high temperature and humidity, water infiltrates the gluey silks of spider orb webs and mobilizes the glycoproteins in the glues (Sahni *et al*., 2011; Stellwagen *et al*., 2014). However, if humidity rises too high, the gluey silks overlubricate and lose their adhesion (Sahni *et al*., 2011). At what specific temperature and humidity this phenomenon occurs differs substantially among different spider species (Opell *et al*., 2018), so it will be very difficult to incorporate into future models but worth attempting.

The dynamic and interactive interplay between temperature and humidity on the stickiness of spider webs, and the variability in responsiveness across species of spider, renders modelling prey retention across changing temperature and humidity particularly complex. A similar dynamic model as that used herein might be utilized to test spiral thread adhesiveness across the same temperature and humidity ranges, but additional parameters such as spiral spacing, spiral thread length, the extensibility, adhesiveness and damping of the spiral threads and any interactions therein, need to be experimentally derived or attained from the literature for inclusion in the model (Guo *et al*., 2018). We did not set out to test the influence of climate change scenarios on web adhesion herein, but we are planning further studies to compare web adhesiveness across a range of spiders using different web architectures and types of sticky spirals.

### Implications for real spiders foraging in changing climates

There are a multitude of practical reasons, which we outlined in the Introduction, why there are not any comparative empirical studies of web performance across the temperature and humidity ranges tested herein. Accordingly, our findings should be considered primarily illustrative but nonetheless important. We expect that the data from which they are founded is robust enough to draw conclusions about the functionality of real-world spider webs under changing climate scenarios. We therefore conclude that humidity has a more direct effect on the performance of spider webs in a changing climate, but temperature could interact with humidity in additional ways to affect prey retention.

We only tested herein whether temperature affected the structural integrity of the spider web and/or the molecular integrity of one material (i.e. MA silk) from which it is manufactured. Another possibility that is worthy of future study is that temperature changes affect spider physiology and thus hamper its ability to produce silks and/or construct a functional web. Indeed, a study on the effect of temperature on spider web building showed the web’s architecture to become affected; with fewer capture spirals with wider spiral spacing found in webs when the external temperature was lowered from 24°C to 12°C (Vollrath *et al*., 1997). Climate change-induced temperature rises might thus affect spider web functionality via changes in web architecture as a consequence of impairment to the spider’s ability to produce silk and/or effectively build a web.

Our study is the first to model plasticity in an EP construct and to extrapolate the results to predict the functionality of the construct and fitness prospects of organisms under climate change scenarios. We concede that our results should not be considered as representative for any spider web or EP construct from any given geographic region. That would require the collection of more specific EP property data for specific species and applying region-specific climate scenarios. Nonetheless, we think that it serves as an important template for future studies that endeavour to make predictions about the impacts of climate change on animals displaying EPs or other complex phenotypic features.
